# *Haematococcus lacustris*: the makings of a giant-sized chloroplast genome

**DOI:** 10.1093/aobpla/ply058

**Published:** 2018-10-01

**Authors:** David Roy Smith

**Affiliations:** Department of Biology, University of Western Ontario, London, Ontario, Canada

**Keywords:** *Corynoplastis*, genome size, green algae, plastid genome, *Volvox*

## Abstract

Recent work on the chlamydomonadalean green alga *Haematococcus lacustris* uncovered the largest plastid genome on record: a whopping 1.35 Mb with >90 % non-coding DNA. A 500-word description of this genome was published in the journal *Genome Announcements*. But such a short report for such a large genome leaves many unanswered questions. For instance, the *H. lacustris* plastome was found to encode only 12 tRNAs, less than half that of a typical plastome, it appears to have a non-standard genetic code, and is one of only a few known plastid DNAs (ptDNAs), out of thousands of available sequences, not biased in adenine and thymine. Here, I take a closer look at the *H. lacustris* plastome, comparing its size, content and architecture to other large organelle DNAs, including those from close relatives in the Chlamydomonadales. I show that the *H. lacustris* plastid coding repertoire is not as unusual as initially thought, representing a standard set of rRNAs, tRNAs and protein-coding genes, where the canonical stop codon UGA appears to sometimes signify tryptophan. The intergenic spacers are dense with repeats, and it is within these regions where potential answers to the source of such extreme genomic expansion lie. By comparing ptDNA sequences of two closely related strains of *H. lacustris*, I argue that the mutation rate of the non-coding DNA is high and contributing to plastome inflation. Finally, by exploring publicly available RNA-sequencing data, I find that most of the intergenic ptDNA is transcriptionally active.

## Teaser

Recent work on the green alga *Haematococcus lacustris* uncovered the largest plastid genome on record: a whopping 1.35 Mb. Here, I take a closer look at this gigantic plastome, comparing it to other large organelle DNAs. I show that the *H. lacustris* plastid coding repertoire is not as unusual as initially thought, representing a standard set of rRNAs, tRNAs and protein-coding genes. The intergenic spacers are dense with repeats, and it is within these regions where potential answers to the source of such extreme genomic expansion lie. By comparing two closely related strains of *H. lacustris*, I argue that the mutation rate of the non-coding plastid DNA is high and contributing to plastome inflation.

## Introduction

Most scientists can probably relate to the following feeling. You are at a conference or reading a new research paper and suddenly overcome by a lust for someone else’s data. You think to yourself: ‘If only I had those results. If only those experiments were mine …’ Recently, I had a terrible bout of data envy. As a long-time organelle genome junky, I regularly explore GenBank for newly sequenced mitochondrial and plastid DNAs (mtDNAs and ptDNAs). This is usually an uneventful task. But the other day, while scanning the newest cohort of plastomes, I was so surprised by what I saw that I nearly spilt my five-dollar Americano onto my five-thousand-dollar laptop. ‘This can’t be right’, I said. ‘A plastid genome with a length of 1352 kb! Did the authors accidentally move the decimal to the right by one?’ I quickly downloaded the genome to a sequence viewer and, sure enough, it was 1.35 Mb, making it the largest ptDNA on record.

**Figure 1.**
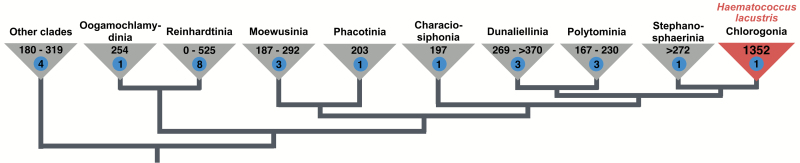
Known plastome size ranges within the Chlamydomonadales. Branching orders and clade names based on Nakada *et al.* (2008). Genome sizes based on published and/or GenBank data.

My genome jealousy only worsened when I realized that the species to which this giant plastome belonged was *Haematococcus lacustris*, a member of the green algal order Chlamydomonadales (Chlorophyceae, Chlorophyta) (Nakada *et al.* 2008) (Fig. 1). For the past decade, my collaborators and I have been documenting extremes in plastome size within this order (Smith *et al.* 2013; Del Vasto *et al.* 2015; Figueroa-Martinez *et al.* 2017; Gaouda *et al.* 2018). Heretofore, the largest ptDNAs that we had uncovered were from the 4-celled *Tetrabaena socialis* (Featherston *et al.* 2016) and the multicellular *Volvox carteri* (Smith and Lee 2010), which, at ~405 and ~525 kb, respectively, are puny compared to the *H. lacustris* ptDNA.

I checked the GenBank entry for the academic article describing this giant genome: Bauman *et al.* (2018). ‘Oh no’, I cried. ‘Please tell me this isn’t what I think it is … anything but a genome announcement’. But, alas, the magnificent *H. lacustris* plastome was written up in *Genome Announcements*, a non-peer-reviewed journal rapidly publishing short, 500-word reports on new microbial chromosome sequences. To an organelle DNA connoisseur like myself, this was equivalent to beer funneling a bottle of 1983 Château Cheval Blanc. [Note, in July 2018 the journal *Genome Announcements* was renamed *Microbial Resource Announcements*.]

My scientific heart was telling me that this plastome should not be laid to rest like this; it deserves to be noticed and appreciated by those in the chloroplast research community. So, here I am, giving the *H. lacustris* ptDNA its dues. In the following pages, I will attempt to explain why this genome is so exceptional and to resolve some of the many questions raised in its initial description. First and foremost, the intent of this commentary is not to criticize Bauman *et al.* (2018). This small team of scientists works for the private company Synthetic Genomics (La Jolla, CA, USA), which is primarily focused on applied research, not organelle genome evolution. We are fortunate that this for-profit company took the time and effort to upload the *H. lacustris* ptDNA to GenBank and write it up in an open-access journal. Now let’s take a closer look at this novel plastome and the species from which it comes.

### Who is *Haematococcus lacustris*?

The genus *Haematococcus*, which is made up of unicellular freshwater biflagellates, was first described over a century and a half ago (Flotow 1844), but is still unknown to most biologists outside of phycology. However, those who keep bird baths in their backyards may have inadvertently cultivated members of this genus, whose lifecycle is well suited to the dry-wet vicissitudes of garden ponds (Almgren 1966). Woe betide to those who do come across a *Haematococcus* bloom for the blood-red colour that typifies most species can be a startling sight (Pocock 1960). This bright pigmentation is caused by the ketocarotenoid astaxanthin, which can accumulate in large quantities within lipid vesicles in the cytoplasm (Collins *et al.* 2011; Ota *et al.* 2018) and is thought to help the alga survive desiccation. Much of the current research on *Haematococcus* centres on the commercial bio-harvesting of astaxanthin for use in the nutraceuticals, cosmetics, food and aquaculture industries (Shah *et al.* 2016), hence why Synthetic Genomics is investing in this green algal lineage.

The *Haematococcus* genus is now understood to be quite diverse, with many different strains and several species having been described (Buchheim *et al.* 2013; Klochkova *et al.* 2013; Allewaert *et al.* 2015). But for the longest time, only one species was formally recognized. The nomenclature of this species is confusing and controversial (Droop 1956; Pocock 1960; Amalgen 1966; Allewaert *et al.* 2015) as it is often referred to using two different names: *H. lacustris* (Girod-Chantrans 1802) Rostafinski and *Haematococcus pluvialis*Flotow 1844. As it currently stands, these two names appear to be synonymous, and based on Nakada and Ota (2016) the correct terminology for the type of *Haematococcus* is *H. lacustris*.

**Figure 2.**
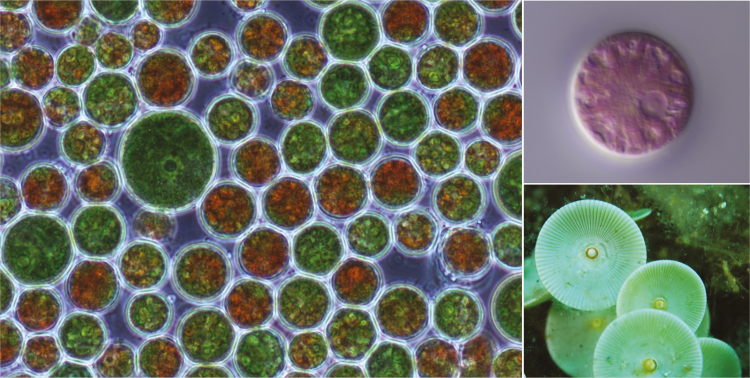
Eukaryotic algae with giant plastomes. Clockwise from left: *Haematococcus lacustris* strain CCMP 3127, which is equivalent to UTEX 2505, the strain used for plastome sequencing (image: National Center for Marine Algae and Microbiota); the unicellular rhodellophycean red alga *Corynoplastis japonica* (image: Sergio Muñoz-Gómez); and the marine unicellular ulvophyte *Acetabularia* sp. (image: Albert Kok).

The epitype of *H. lacustris*, originally collected by F. Mainx in the former Czechoslovakia and then isolated by E. G. Pringsheim, is cryopreserved and available from the Microbial Culture Collection at the National Institute for Environmental Studies, Japan, under strain number NIES-2264. Bauman *et al.* (2018) did not use this strain for plastid genome sequencing. Instead, they employed *H. lacustris* strain UTEX 2505 (Fig. 2), the history of which I could not find. Phylogenetic analyses of UTEX 2505 suggest that it is very closely related to UTEX 16, a descendant of NIES-2264 (Buchheim *et al.* 2013).

### Early indications of a king-sized plastome

It really should come as no surprise that the *H. lacustris* plastome was found to be so large. As part of a phylogenetic survey of chlorophyceans, Lemieux *et al.* (2015) generated a draft plastid genome assembly of *H. lacustris* strain SAG 34-1b (equivalent to NIES-2264). This assembly, which was based on Illumina sequencing of total cellular DNA, was highly fragmented, comprising 94 contigs (GenBank accessions KT625205–98), ranging from 1.2 to 13.8 kb. Nevertheless, the accumulative length of these contigs (302.8 kb) was indicative of a particularly large ptDNA, but precisely how large remained to be determined.

The sequencing effort of Lemieux *et al.* (2015) provided a clear, near-complete picture of the *H. lacustris* ptDNA coding content. It identified the standard set of proteins and rRNAs normally encoded in a chlamydomonadalean plastome, as well as 26 tRNAs, which is slightly less than a typical plastid tRNA cohort, but presumably the missing ones were not captured in the assembly. Together, the ptDNA contigs contained 26 introns, which is a lot for a green algal plastome, but not unheard of (Del Vasto *et al.* 2015). Perhaps the most salient feature of the data was the exceptionally high repeat content of the intergenic regions, which obviously played havoc on the short-read assembly. It is no surprise, then, that it took a long-read sequencing approach to finally piece together this prodigious plastome.

### Updating the annotation

Bauman *et al.* (2018) sequenced and assembled the 1.352 Mb *H. lacustris* ptDNA using PacBio single-molecule real-time (SMRT) sequencing. Their *de novo* plastome assembly included 91000 subreads, with a mean length of 8900 bp, and yielded a single complete ptDNA contig with >500× coverage. The genome is circularly mapping, but probably has a more complicated architecture (Bendich 2004), and contains a pair of long inverted repeats, which is a characteristic of most ptDNAs (Turmel *et al.* 2017). As of the 1 September 2018, the annotation of the *H. lacustris* plastome (GenBank accession NC_037007) is unfinished and contains inaccuracies (Gaouda *et al.* 2018). For example, Bauman *et al.* (2018) characterized 125 protein-coding regions, but only 12 tRNAs and no rRNAs, despite the fact that Lemieux *et al.* (2015) had already characterized 26 tRNAs and the complete rRNAs from *H. lacustris*. Moreover, many of the annotated protein-coding regions are hypothetical or fragmented, and there are also instances of proteins not ordinarily found in plastids, such as nicotine oxidoreductase. When the *H. lacustris* ptDNA sequence is run through a standard organelle genome annotation pipeline, such as GeSeq (Tillich *et al.* 2017) or MFannot (Lang *et al.* 2007), a standard and complete stock of plastid protein-coding genes and functional RNAs can be identified, including 31 tRNAs.

Some of the protein-coding genes in the *H. lacustris* ptDNA appear, at first glance, to be fragmented. For example, the *ftsH* gene in the Bauman *et al.* (2018) annotation is distributed across five distinct (but consecutive) open reading frames (ORFs). However, the Lemieux *et al.* (2015) annotation of *ftsH* shows an intact gene. The reason for these discrepancies is that *ftsH* (and other genes in the *H. lacustris* ptDNA) contains multiple occurrences of the internal stop codon UGA. In their GenBank data, Lemieux *et al.* (2015) have marked these premature UGAs as sense codons for tryptophan, implying that the *H. lacustris* plastome employs a non-standard genetic code, but they do not elaborate on this in the manuscript.

To provide further support of a non-standard code in the plastid of *H. lacustris*, I explored 16.5 Gb of publicly available RNA-sequencing (RNA-seq) data from two different isolates of *H. lacustris* (strain CCAC 0055, Culture collection of Algae University of Cologne; and strain IOCAS 712, Institute of Oceanology, Chinese Academy of Sciences), which were generated as part of the 1000 Plants Project (Matasci *et al.* 2014) and a study on astaxanthin induction (Gao *et al.* 2015) (Short Read Archive accessions ERX2100117–8 and SRX1136554–9). Using the Geneious v10.2.4 read mapper (medium sensitivity; default settings), I mapped 15.3 million reads from these data onto the *H. lacustris* ptDNA, covering ~90 % of the genome (discussed in detail later) **[see****Supporting Information—Fig. S1****]**. Genes with internal stop codons, including *fstH* and *ycf1*, had high coverage (>50×), and there were no obvious drops in coverage preceding or proceeding internal UGAs and no indications of RNA editing. All of this is consistent with UGA signifying tryptophan in a subset of the *H. lacustris* plastid genes. Such a deviation is not unprecedented. In the ptDNA of the green algae *Boodlea composita* (Ulvophyceae) and *Jenufa minuta* (Chlorophyceae), UGA also appears to have a double meaning, representing tryptophan in some genes and a stop codon in others (Del Cortona *et al.* 2017; Turmel and Lemieux 2017).

The *H. lacustris* and *B. composita* plastomes have another interesting similarity: both have unusually high GC contents for organelle DNAs (50 and 57 %, respectively); in fact, *H. lacustris* has the sixth highest GC composition yet recorded in a plastid genome. Despite these similarities, and the fact that the *B. composita* ptDNA might also be quite large (Del Cortona *et al.* 2017), there does not appear to be any obvious relationship between GC content and genome size or non-canonical genetic codes in organelle systems (Smith 2012).

### Big compared to what?

Whether a genome is big or small is a relative concept. Of the more than 2800 complete plastome sequences in GenBank, 98 % are under 200 kb and harbour modest amounts (<50 %) of non-coding DNA. But there are a few algal lineages whose members can have much larger ptDNAs with an abundance of non-coding nucleotides (Moreira and López-García 2017; Smith 2018). The closest rivals to the *H. lacustris* plastome in terms of size are the ptDNAs of the unicellular rhodellophycean red algae *Corynoplastis japonica* (1.13 Mb) (Fig. 2) and *Bulboplastis apyrenoidosa* (0.61 Mb) (Muñoz-Gómez *et al.* 2017). The former currently holds the record for the most introns found in an organelle genome (311), followed in second place by none other than *B. apyrenoidosa* (220). The unrivalled proliferation of introns in these two genomes contrasts the mode of expansion in *H. lacustris*, which, as noted earlier, is not particularly intron dense.

The ptDNA of the unicellular marine green alga *Acetabularia acetabulum* (Ulvophyceae) (Fig. 2) might be even bigger than that of *H. lacustris* and its rhodellophycean rivals. Initial ptDNA size estimates for this alga (and its close relative *Acetabularia cliftonia*), based on electron microscopy (Green 1976), kinetic-complexity analyses (Padmanabhan and Green 1978) and restriction digest experiments (Tymms and Schweiger 1985), suggested a length of up to 2 Mb. The genome, however, has not been completely sequenced. de Vries *et al.* (2013) partially assembled the *A. acetabulum* ptDNA (using 454 sequencing data) into 63 contigs, totalling ~350 kb and containing 39 full-length genes and a modest number of large introns. But the short-read data were not enough to bridge the elongated intergenic regions, meaning its exact size is unknown.

The green algal order to which *H. lacustris* belongs—the Chlamydomonadales—is home to a number of species with plastomes in excess of 300 kb (Fig. 1), such as *Carteria cerasiformis* (Lemieux *et al.* 2015) and *Dunaliella salina* CONC-001 (Del Vasto *et al.* 2015). Expansion appears to be particularly prevalent within the volvocine line (Gaouda *et al.* 2018), where there exists more than a 2-fold variation in ptDNA size and some of the largest, most repeat-dense plastomes yet sequenced (Fig. 1). Remarkably, plastome inflation within this order is not limited to photosynthetic species: the free-living, unicellular chlamydomonadalean *Polytoma uvella* harbours the largest ptDNA of any colourless plant or algae (~230 kb) (Figueroa-Martinez *et al.* 2017). Why these various plastomes have been pushed to such extremes in size is still not fully understood.

### Why so large?

Much of our understanding of organelle genome expansion comes from land plant mtDNAs, which can be as large as 11.3 Mb (Sloan *et al.* 2012). For example, Lynch *et al.* (2006) eloquently argued that mtDNA inflation in plants is a consequence of a low mutation rate, which in turn reduces the burden of harbouring excess non-coding DNA. Such a model is consistent with the low levels of synonymous site substitution regularly observed in land plant mitochondria (Wolfe *et al.* 1987; Richardson *et al.* 2013). More recently, Christensen (2013, 2017) found that the intergenic sites of angiosperm mtDNAs can have much higher rates of substitution (and insertion-deletion) than synonymous sites, suggesting that mitochondrial genome expansion might result from error-prone DNA repair mechanisms within non-coding regions, such as break-induced replication (BIR).

To get a cursory sense of the underlying plastid mutational spectrum in *H. lacustris*, I mapped the 94 ptDNA contigs of Lemieux *et al.* (2015) to the complete plastome sequence of Bauman *et al.* (2018). Again, these two data sets come from different isolates of *H. lacustris* (strains SAG 34-1b and UTEX 2505, respectively). The Geneious mapper matched 84 contigs to the reference genome, giving 255 kb (19 %) of coverage, including 88 kb of coding and 167 kb of non-coding ptDNA. Levels of polymorphism between SAG 34-1b and UTEX 2505 were low in the coding regions (nucleotide diversity < 0.005). Of the 60 plastid protein-coding genes that aligned, 41 were identical, and the remaining 19 all showed >97 % pairwise identity. In total, the aligned protein-coding regions contained 448 indels (0.3 %). Conversely, I found large numbers of polymorphisms and indels in the repeat-rich non-coding regions. The pairwise nucleotide diversity of the 167 kb of aligned non-coding ptDNA was 0.17, containing 7964 indels (2.3 %). These findings parallel the trends in angiosperm mtDNAs (Christensen 2013) and suggest that the repeat-riddled non-coding regions of the *H. lacustris* plastome are prone to errors, particularly indels. More detailed sequence analyses of *H. lacustris* will need to be carried out to support these suggestions, especially because sequences containing similar but distinct repeats could have been condensed into a single region, either at the assembly (Lemieux *et al.* 2015) or mapping stages, which in turn could have inflated the observed levels of intergenic nucleotide diversity. Nevertheless, these preliminary data and their similarities with the enormous mtDNAs of land plants are intriguing.

It has also been suggested that there might be a positive relationship between plastid genome size and cell size in plastid-bearing protists (Smith 2017). Indeed, algae with tiny cells often have miniature ptDNAs, such as the prasinophyte *Ostreococcus tauri*, which is ~0.8 μm in diameter (Courties *et al.* 1994) and has a ptDNA of only 71.7 kb (Robbens *et al.* 2007). Likewise, the unicellular *A. acetabulum* is so gargantuan that it can be seen with the naked eye (1–10 cm) (Fig. 2), and, as already noted, boasts a very big plastome. The cell size of *C. japonica*, although not as extraordinary as *A. acetabulum*, is also quite large (18–33 µm in diameter) (Yokoyama *et al.* 2009), and an order of magnitude larger than that of *O. tauri*. In this context, it is noteworthy that *H. lacustris* is relatively big. Vegetative cells are typically 29–39 μm long and 18–32 μm wide, and aplanospores are usually around 27–58 μm in diameter (Nakada and Ota 2016), further supporting the idea that large plastomes frequently occur within large cells.

### Pervasive transcription of non-coding ptDNA

More and more studies are uncovering pervasive genome-wide (or near genome-wide) transcription of organelle DNAs (Mercer *et al*. 2011; Zhelyazkova *et al.* 2012), including those from a wide range of plants and algae (Shi *et al.* 2016; Sanitá Lima and Smith 2017). With some exceptions (Wu *et al.* 2015), even very large organelle genomes, such as the mtDNA of *Cucurbita pepo* (~982 kb), were found to be almost fully transcribed, implying that large amounts of non-coding organelle RNA can be generated in organelle systems (Sanitá Lima and Smith 2017). However, a lack of data has heretofore prevented researchers from studying pervasive transcription of large plastomes (>250 kb). *Haematococcus lacustris* is an ideal candidate for exploring such a topic, given its gigantic ptDNA and the fact that it has been the focus of multiple transcriptome sequencing efforts (Matasci *et al.* 2014; Gao *et al.* 2015).

By mapping publicly available RNA-seq reads from *H. lacustris* strains CCAC 0055 and IOCAS 712 (strains, data and methods described earlier in text) to the *H. lacustris* plastome, I was able to cover ~90 % of reference sequence with one or more reads **[see****Supporting Information—Fig. S1****]**. The read coverage was highest in the coding regions (>50×), dropping off significantly in the intergenic regions (average ~10×). Nevertheless, more than 900 kb of non-coding ptDNA was represented in the RNA-seq data, indicating that, like other organelle DNAs, the *H. lacustris* ptDNA can exhibit pervasive transcription. Further work will be needed to substantiate these findings. One should keep in mind that genomic DNA (local or foreign) can persist in RNA-seq libraries even after treatments to eliminate it (Haas *et al.* 2012), and there is always the potential of mistaking nuclear-located, plastid-like sequences (NUPTs) as genuine plastid DNA/RNA, but the rate of plastid-to-nucleus DNA transfer in *H. lacustris* is presumably very low given that it only has a single plastid per cell (Smith *et al.* 2011). As it currently stands, the *H. lacustris* ptDNA appears to be a veritable RNA machine.

The available RNA-seq data from *H. lacustris* might also be a good source for mining polymorphisms and exploring nucleotide diversity between strains of *H. lacustris*. For example, CCAC 0055 is a distinct geographical isolate of *H. lacustris*, collected from a rain-water reservoir near Cologne, Germany, in 1990. That said, I found that mapping the short-read RNA-seq data to the intergenic regions was complicated by the large numbers of near-identical repeats that have spread throughout the plastome. More accurate understanding of diversity of the intergenic DNA might have to come from long-read data, and preferably using a *de novo* assembly approach to ensure a better resolution of the repeat regions. With the growing importance of *H. lacustris* as an industrial alga, there will likely be a lot of sequencing data arriving to GenBank in the coming months and years—which is music to my ears because this unicell with its behemoth of a plastome surely has a lot more to teach us about organelle genome evolution.

## Sources of Funding

This work was supported by a Discovery Grant to D.R.S. from the Natural Sciences and Engineering Research Council of Canada.

## Supporting Information

The following additional information is available in the online version of this article—

**Figure S1.** Log-scale RNA-seq coverage of the *Haematococcus lacustris* plastid genome.

supplementary Figure S1
