# Supplementary material for: Haematococcus lacustris: the makings of a giant-sized chloroplast genome
Source: AoB Plants. 2018 Oct 1;10(5):ply058. doi: 10.1093/aobpla/ply058 (PMC6205361; doi:10.1093/aobpla/ply058)
Supplement: supplementary Figure S1 [file ply058_suppl_figure_s1.pdf]

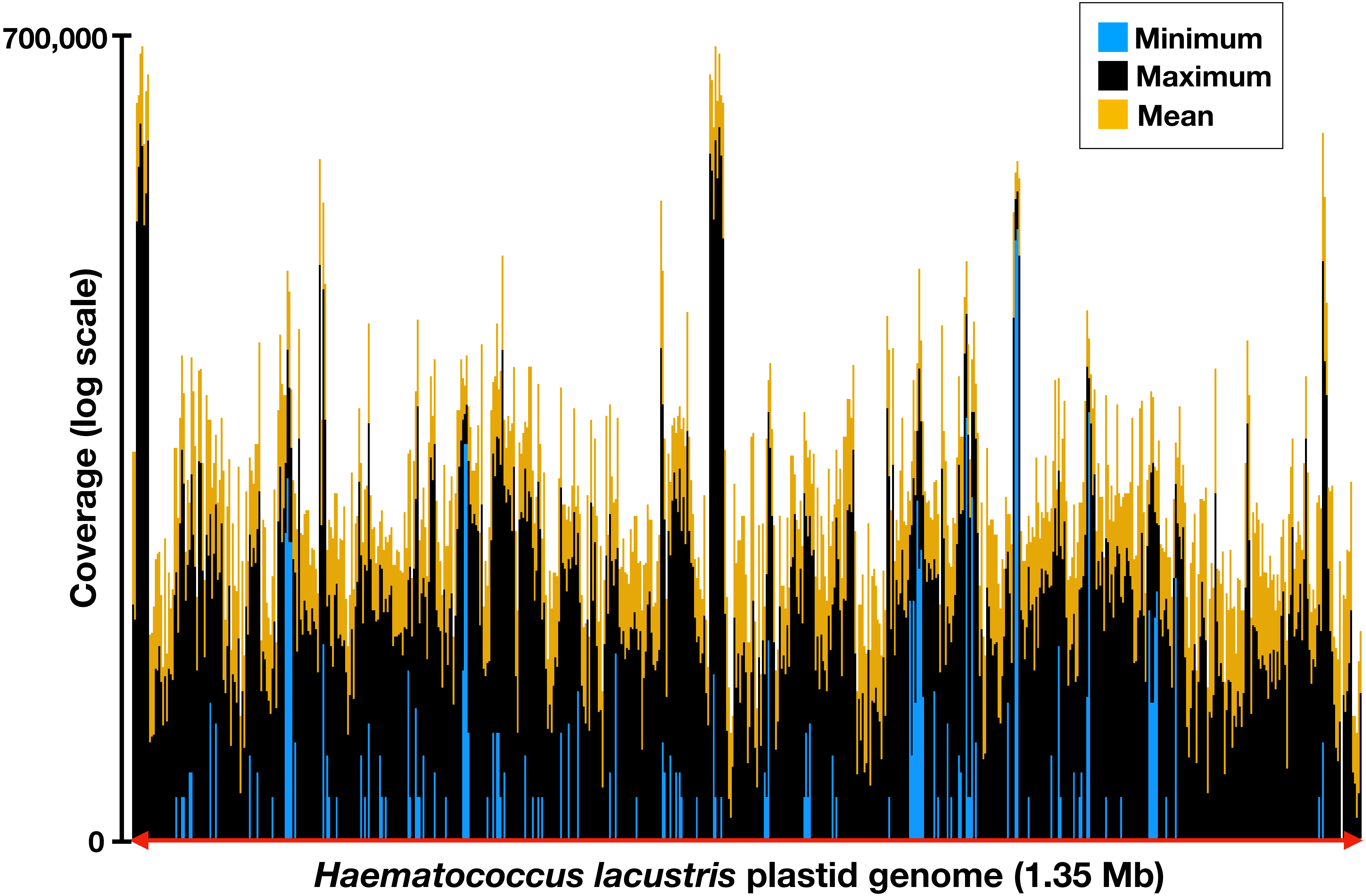

Figure S1. Log-scale RNA-seq coverage of the *Haematococcus lacustris* plastid genome: log(coverage + 1)/log(maximum coverage + 1).
